# Factors influencing the efficiency of cocoa farms: A study to increase income in rural Indonesia

**DOI:** 10.1371/journal.pone.0214569

**Published:** 2019-04-04

**Authors:** Effendy, M. Fardhal Pratama, Rustam Abdul Rauf, Made Antara, Muhammad Basir-Cyio, Mahfudz, Muhardi

**Affiliations:** 1 Department of Agriculture Economics, Agriculture Faculty of Tadulako University, Palu, Indonesia; 2 Department of Agroecotechnology, Agriculture Faculty of Tadulako University, Palu, Indonesia; University of Tehran, ISLAMIC REPUBLIC OF IRAN

## Abstract

Indonesia is the fifth largest cocoa-producing country in the world, and an increase in cocoa farming efficiency can help farmers to increase their per capita income and reduce poverty in rural areas of this country. This research evaluated the efficiency of Indonesian cocoa farms using a non-parametric approach. The results revealed that the majority of cocoa farms are operated relatively inefficiently. The average technical and allocative efficiencies (0.82 and 0.46, respectively) of these cocoa farms demonstrated that there is potential for improvement. The potential cost reductions range from 36 to 76%, with an average of 60%, if farmers practice efficiently. The technical and allocative efficiencies and cocoa farm economies are affected by the use of quality seeds, organic fertilizers, frequency of extension and training of farm managers, access to bank credit and the market, the participation of women, and the farm manager’s gender. An increase in the output would increase farmers’ income and reduce poverty in rural areas. This research suggests that the availability of extension and training provided to farmers as well as support for women farmer groups should be increased. Credit programs are also important for cocoa farmers, so policymakers should develop programs that make production credit more accessible for farmers, especially through cooperatives and banks.

## Introduction

Agriculture plays an important role in the reduction of poverty and starvation in Asia [1]. It also helps to reduce poverty by generating employment and income [2]. Poverty eradication is currently the main objective in Indonesia. To realize this goal, cocoa farms have become a priority area [3].

Indonesia is the fifth largest cocoa producer in the world, after Côte d’Ivoire, Ghana, Ecuador, and Nigeria. In Asia and Oceania, Indonesia is ranked as the highest producer of cocoa, with an annual production of approximately 240,000 tonnes (source: ICCO 2018), followed by Papua New Guinea, which has an annual production of only approximately 40,000 tonnes [4]. The government has formed the target that, within the next five years, Indonesia should become the world’s largest cocoa producer.

Historically, cocoa has played an important role in supporting Indonesia’s economic development as an export commodity. However, cocoa production has recently encountered various problems. These problems are manifold: plant pests and diseases, such as the cocoa pod borer, vascular streak dieback, and cocoa black pod disease; a decrease in productivity; poor quality of cocoa beans and old cocoa plants; and sub-optimal management of land resources [3]. To overcome these problems, the government, through the National Cocoa Quality and Production Promotion Movement (referred to as Gernas in Indonesia), has promoted the use of high-yielding clones whose output ranges from 1.80 to 2.75 t ha^–1^ [5]. However, in achieving the target, cocoa farmers face several obstacles.

Commonly, agricultural households in Indonesia have relatively low levels of education and technological adoption and use resources inefficiently [6,7]. This leads to high production costs and the loss of cost advantages in relation to cocoa exports [8]. However, with an increase in efficiency, Indonesia can boost its comparative advantage in cocoa production and export marketing. Improved efficiency may lead to increase the productivity (which is presently 0.88 t ha^–1^ [9], potentially from 1.8 to 2.75 t ha^–1^), which will enable cocoa farmers to meet domestic and international demands from countries such as China, Germany, India, Japan, Malaysia, the Netherlands, Singapore, Thailand, and the United States [9].

However, to boost the comparative advantage, Indonesian cocoa producers must achieve higher technical efficiency (TE = 1 shows that cocoa farming is technical efficient). Changes in TE will impact cocoa productivity. The use of efficient resources (inputs) will increase agricultural output and income [10–14]. Therefore, there is a need to analyze the relationship between the input, farm characteristics, socio-economic factors, and efficiency involved in cocoa production [15–17]. This research could serve as the basis of policies promoting cocoa efficiency at the farm level. The objectives of this research were to evaluate the efficiency of cocoa farms and to suggest some priority areas for policy interventions designed to increase the efficiency of cocoa production, which, in turn, could increase income and reduce poverty in rural Indonesia.

## Materials and methods

### Research areas and sampling methods

The region of Sulawesi, one of the six cocoa-producing regions in Indonesia, was selected as the research area. It is the largest cocoa-producing region, both in terms of area (ca. 60% of the national area) and production (ca. 70% of national production) (Table 1).

**Table 1:** Regions of cocoa plantations (smallholders) in Indonesia in 2014.

No.	Region	Area		Production (tonnes)
		ha	% of national	
1	Sulawesi (Celebes)	975,821	59.61	456,965
2	Sumatera	400,038	24.44	125,176
3	Java	58,433	3.57	13,928
4	NTT + NTB + Bali	70,075	4.28	15,639
5	Kalimantan (Borneo)	35,012	2.14	8,797
6	Maluku + Papua	97,498	5.96	31,113
	Total	1,636,877	100.00	651,618

Source: Data from Ditjenbun [9], after processing.

Four out of the thirteen districts were purposefully selected for this research. In each district, two villages were randomly selected for the survey (Table 2).

**Table 2:** The research areas and their characteristics.

District	Villages	Sample size (household heads)	Socio-economic conditions
Donggala	Watatu	87	Access to good roads, market facilities, and extension services
	Salumpaku		
Parigi Moutong	Kotaraya	98	
	Kayu Agung		
Sigi	Sejahtera	144	
	Tongoa		
Poso	Lape	95	
	Kilo		
Total		424	

In total, 424 household heads (HH) of cocoa farmers were selected randomly from all cocoa farmers who produced cocoa beans in the sample area for the purpose of answering the questionnaires. The data were analyzed anonymously; for this purpose, the questionnaire was created without a name line for the respondents. During the interviews, the background of the research was explained to the respondents, and they were also told that there was no pressure on them to participate. The respondents were cooperative in providing information.

This research employed a cross-section of data, comprising both qualitative and quantitative data. Questionnaires were used in this research for data collection; the questions concerned education, respondents’ land ownership, the types of seeds they used, their use of organic fertilizer, access to credit, access to market, and farm managers’ gender (elements of qualitative data). The other factors studied were age, income, land area, cocoa production, cost of cocoa production with regard to various inputs, extension, and training access (elements of quantitative data), as well as other variables required for this analysis. The data collection process lasted from January to April 2016.

### Analysis of efficiency using a non-parametric approach

Efficiency is defined as the maximum ratio of weighted output against weighted input for each decision-making unit (DMU) [18]. In general, two methods are used to measure efficiency: parametric and non-parametric approaches. The non-parametric method employs a deterministic approach (linear programming), which means that the most efficient producers are identified in the observation of each DMU. This method was first proposed by Farrell [19] and was described in entirety by Charnes et al. [20]; it later came to be known as data envelopment analysis (DEA). DEA refers to non-parametric methodology based on linear programming (LP) which is used to analyze the functions of production through mapping frontier production [21]. This approach does not require any particular functional form and does not impose an a priori parametric restriction on the underlying technology. In addition, the non-parametric approach can be used for technology that involves multiple inputs and outputs; further, it can be used to estimate the technical, allocative, economic, and scale efficiencies. Numerous studies have analyzed the efficiency of farms using DEA [22–29]. However, research on perennial crops and farm efficiencies (cocoa) is limited.

This research evaluated the efficiency of cocoa farms in terms of the output using DEA, assuming constant returns to scale (CRS) if operating at the optimal scale. Assume the number of DMUs is q; then, the cocoa farms would produce one type of output using different inputs (p). Here, *Y_i* represents the output production, *X*_*i* represents the input vector (p × 1), Y represents the output vector (1 × q), and X represents the input matrix (p × q) of the DMU. Then, the problem can be stated as follows:

MinZ_i

s.t.−Y_i+Yλ≥0,

Z_iX_i−Xλ≥0,

λ≥0 (1)

Here, *Z*_*i* represents the TE score of the i^th^ DMU under the condition of CRS, and *λ* is a vector q x 1. If the optimal value of Z_i is equal to one, then the DMU under evaluation will be on the weak frontier and will achieve TE equal to one under the CRS assumption of the production technology. If Z_i<1, then the technically efficient production cost of the *i*^*th*^ DMU is given by $W_{-}^{'}i\left(Z_{-}iX_{-}i\right)$ for CRS models, where $W_{-}^{'}i$ represents the vector of input prices. TE refers to the ability of farms to produce the optimum output through the use of certain inputs or their ability to produce some output levels from a minimum number of inputs with a specific technology.

The cost minimization of the input DEA (Eq 2) with assuming CRS, defined in the LP (Eq 1), was used to obtain TE:

$$Min\;W_{-}^{'}iX_{-}i,$$

$$s.t.-Y_{-}i+Y\lambda \geq 0,$$

$$X_{-}i-X\lambda \geq 0,$$

λ≥0 (2)

The cost efficiency model written in Eq 2 has been simplified in a paper entitled “A simplified version of the DEA cost efficiency model” [30], where the cost minimization or economic efficiency (EE) of the input vector *i*^*th*^ for DMU is, *X_i* the vector of input prices is $W_{-}^{'}i$, and the level of output is $Y_{-}i$. Overall, the EE score of the DMU for the *i*^*th*^ farm is calculated as the ratio of the minimum cost, where the cost was observed [19] and was comparable to the scores of the EE (Eq 3); if EE = 1 is considered economically efficient, EE <1 indicates EE was not attained.

$$EE=\frac{W_{i}^{'}X_{i}^{*}}{W_{i}^{'}X_{i}}$$ (3)

The superscript star stands for optimality.

The allocative efficiency index is calculated with Eq (4).

$$AE=\frac{EE}{TE}$$ (4)

Allocative efficiency (AE) signifies the ability of the farms to equate the value of the marginal product with the marginal cost. If AE = 1, it implies that the farms have reached an efficient price; an AE value smaller than one implies that farms are inefficient in terms of price, so the cost must be minimized, while an AE > 1 implies that farms are yet to reach price efficiency. Among these three measurements (TE, AE, and EE), TE and EE can take a value range of 0 to 1, while AE can be in the range 0 to 1 and > 1, where a value of 1 signifies complete efficiency.

Both models (1) and (2) are under CRS production technology. The VRS versions of these models are obtained by imposing the equality of the sum of the components of lambda vector with one

### Tobit analysis

In this research, the variable obtained from the DEA procedure was mostly employed as the dependent variable in further analysis between socio-economic resources and an efficiency unit [31–34]. After the calculation of efficiency using DEA, this research aimed to ascertain the determinant factors in cocoa farms’ efficiency (farm variables related to technology and socio-economic characteristics). Because the efficiency value is limited between zero and one, this research employed the Tobit regression model using the maximum likelihood (ML) approach [35].

$$EE_{i}^{*}=\alpha_{0}+\sum_{j=1}^{k}\alpha_{j}K_{i}{}_{j}+\mu_{i},\;\mu_{i}\sim ind\left(0,\sigma^{2}\right)$$ (5)

$EE_{i}^{*}$ represents a dependent variable that represents the economic efficiency scores for the *i*^*th*^ DMU, expected from *EE*_*i*_ of the DEA model; *α*_0_ and *α*_*j*_ are the estimated parameters; *K*_*ij*_ represents the independent variable associated with cocoa farms as written in the "Variables’ specification" section in Eq (7); *μ*_*i*_ constitutes an error term which is independently and normally distributed (0,*σ*^2^).

### Variable specification

The cocoa production function employed to the specifications of the variables in Eqs (1)–(4) is represented as follows:

$$Y_{i}=\beta_{0}+\beta_{1}X_{1}+\beta_{2}X_{2}+\beta_{3}X_{3}+\beta_{4}X_{4}+\beta_{5}X_{5}+\beta_{6}X_{6}+e_{i}$$ (6)

where

Y = production of cocoa;
X_1_ = land;
X_2_ = chemical fertilizer;
X_3_ = labor;
X_4_ = cost of pesticide;
X_5_ = cost of pruning; and
X_6_ = cost of sanitation.

The determinants of cocoa farms’ efficiency (farm variables related to technology and socio-economic characteristics) in Eq (5) are represented as follows:

$$EE_{i}=\alpha_{0}+\alpha_{1}K_{1}+\alpha_{2}K_{2}+\alpha_{3}K_{3}+\alpha_{4}K_{4}+\alpha_{5}K_{5}+\alpha_{6}K_{6}+\alpha_{7}K_{7}+\mu_{i}$$ (7)

where

EE_i_ = DEA efficiency for DMU-i
K_1_ = type of seed (dummy)
      0 = for local seeds (seeds were obtained from farmers’ gardens)
      1 = for quality seed (improved (hybrid) seed and clonal cuttings for grafting released by the Ministry of Agriculture)
K_2_ = the use of organic fertilizers (dummy)
      0 = for those who do not use organic fertilizers
      1 = for those who use organic fertilizers
K_3_ = extension and training (number of visits)
K_4_ = access to credit (dummy)
      0 = for non-bank
      1 = for bank
K_5_ = access to market (dummy)
      0 = for those who do not have access to market
      1 = for those who have access to market
K_6_ = participation of women (number)
K_7_ = gender of farm manager (dummy)
      0 = for men
      1 = for women

Some reasons for the inclusion of technological and socio-economic characteristics in the models for efficiency analysis have been presented here. The type of seeds used had a strong effect on the agricultural production. The type of grafting (side grafting and other grafting) was found to affect cocoa production in the Parigi Moutong Regency [36]. The cacao seeds used by farmers in the research area are local and quality seeds. Local seeds are obtained from farmers’ gardens, and quality seeds are improved (hybrid) seed and clonal cuttings for grafting released by the Ministry of Agriculture. The seed output is high, and seeds are resistant to pests and diseases, for example, Sulawesi 1 and Sulawesi 2 are clones, derived from grafts rather than seed [5]. Therefore, we included the seed quality as a dummy variable. The use of organic fertilizers was beneficial for increasing soil fertility. Organic fertilizers affect productivity and the quality of agricultural production [17,37,38]. Therefore, in our model, we introduced organic fertilizers as a dummy variable. Extension and training activities played a role in the spread of technology among farmers. The Central Sulawesi plantation office conducted extension and training for farmers on a bimonthly basis. In addition, assistance was provided so that the farmers could effectively use the available technology. The technology provided included side-grafting techniques, pruning, sanitation, chemical and organic fertilization, pest and disease control, and fermentation. The extension affected farmers’ adoption of agricultural technology [6,39,40]. Therefore, we included extension and training in the model.

Due to a lack of access to financial resources, farmers often rely on informal sources of credit (moneylenders, relatives, and friends), who charge higher interest rates than the bank. The credit affects the usage of agricultural inputs, as farmers can buy more agricultural machinery with greater credit [41]. Therefore, we included farmers’ access to credit as a dummy variable. Small farmers are further restricted by limited market access. Market access affects cocoa prices at the farm level [16]. Therefore, we included market access as a dummy variable. Cocoa farming is extremely labor intensive, and women form the harvest and post-harvest labor source in Indonesia. Gender inequality was also found to limit economic growth. Thus, it was important to include gender in the efficiency analysis of agricultural production [42]. Therefore, we introduced two gender-related indicators in the model (participation of women and the gender of farm managers).

The collection of data, i.e., Y, X_1_–X_6_, K_1_–K_7_, was completed using a semi-structured questionnaire. For the analysis of EE and AE, input costs such as land, chemical fertilizer, labor, pesticides, pruning, and sanitation were measured in rupiah (IDR 13,100 = 1 USD in January 2016) on the basis of the prices paid by the farmers. The price of the output was the price received by the farmers for the sale of cocoa. The land rental prices prevalent in the research area were considered as the cost of land. The type of seeds and organic fertilizers used, credit and market access, and farm managers’ gender were considered dummy variables (Eq 6). The household members responsible for making decisions pertaining to the cocoa farms were considered farm managers. Extension and training were measured in terms of the presence of the farm manager in the activity, while women’s participation was measured in terms of their contribution to cocoa-farming activities.

## Results and discussion

### Description of research variables

A description of the variables used in this research is presented in Table 3.

**Table 3:** Description of research variables.

Variable	Units	Mean	Std. Deviation
Output	kg farm^–1^ year^-1^	1,590.53	718.44
Output	kg ha^-1^ year^-1^	971.45	272.52
Land	ha farm^–1^	1.63	0.56
Chemical fertilizer	kg farm^–1^ year^-1^	890.33	355.21
Labor	man-days farm^–1^ year^-1^	206.79	93.67
Cost of pesticide	IDR farm^–1^ year^-1^	728,755	722,915
Cost of pruning	IDR farm^–1^ year^-1^	901,651	1,051,183
Cost of sanitation	IDR farm^–1^ year^-1^	1,235,495	1,220,029
Type of seed	% quality seed	44.30	49.70
Use of organic fertilizer	% organic fertilizer	40.60	49.20
Extension and training	Number of visits year^-1^	4.40	1.98
Access to credit	% access to bank	52.80	50.00
Access to market	% access to market	52.60	50.00
Participation of women	Number of women	0.40	0.19
Gender of cocoa farm managers	% women	36.3	48.10

The average size of the cocoa farms was 1.6 ha, and the main costs involved in cocoa production included chemical fertilizers, labor, pesticides, pruning, and sanitation. Fewer than 50% of the farmers studied used quality seeds and organic fertilizer. The average extension and training of farm managers was less than 5 times per year. A total of 53% of farmers accessed bank facilities for credit, while fewer than 55% of farmers had access to cocoa markets. The contribution of women constituted 40% of the total labor input, and farms that were managed by women totaled 36%, demonstrating that women’s role in cocoa farms is substantial.

### Estimation of cocoa farm efficiency

Ordinary least squares (OLS) was employed to identify the real conditions of farmers involved in cocoa production. The OLS estimation results of this research are presented in Table 4.

**Table 4:** Parameter estimation based on the OLS method for cocoa farms.

Model	Coefficients	Std. error	Rank
Constant	3.88	0.26	
lnLand	0.13**	0.05	5
lnLabor	0.14***	0.05	4
lnChemical fertilizer	0.29***	0.04	1
lnPesticide	0.27***	0.05	2
lnPruning	0.14***	0.02	3
lnSanitation	0.11***	0.01	6
Sum of elasticities	1.08		
Adjusted R Square	0.91		

Note:

*** Significant at 1%,

** Significant at 5%

Table 4 presents all the independent variables that were significant in determining the cocoa output. These results are consistent with previous findings [8] but differ in the estimation parameters. The sum of the elasticities is 1.08. An elasticity approaching one indicates CRS in cocoa production. Chemical fertilizers showed the highest elasticity in cocoa output, whereas sanitation demonstrated the lowest elasticity. Pesticides were ranked second in terms of elasticity, as cocoa plants are often attacked by pests and diseases.

The cocoa farm efficiency scores were analyzed using the DEAP 2.1 program [43]. The cocoa farms’ technical, allocative, and economic efficiency scores assessed with the CRS and VRS approaches are presented in Table 5.

**Table 5:** Scores of technical, allocative, and economic efficiencies scores for the DEA model.

Efficiency score	TE		AE		EE		SE
	CRS	VRS	CRS	VRS	CRS	VRS	
	%		%		%		%
< 4.00	0.71	0.00	40.80	43.16	58.02	52.59	0.71
4.00–0.49	3.54	0.00	16.98	16.27	14.62	15.57	0.47
0.50–0.59	4.25	4.72	14.86	14.15	11.32	12.03	2.12
0.60–0.69	9.43	4.01	11.79	11.79	6.84	7.31	4.72
0.70–0.79	25.24	15.09	8.02	7.31	4.95	5.66	8.96
0.80–0.89	20.28	12.03	5.43	4.01	2.83	3.77	10.38
≥ 0.90	36.56	64.15	2.12	3.30	1.42	3.07	72.64
Mean efficiency	0.82	0.90	0.46	0.45	0.38	0.41	0.92

Note: TE = overall TE, AE = allocative efficiency, EE = economic efficiency, SE = scale efficiency

Table 5 demonstrates that there was a difference between the TE, AE, and EE scores based on the CRS and VRS approaches. The farmers applied a number of different technologies (e.g., fertilizer), and most of these were inefficient. To reduce inefficiency, farmers need to adopt technologies in accordance with the government’s advice and to improve farms’ managerial aspects through non-formal education (e.g., extension). The average TE and EE scores based on the VRS approach were higher than those obtained with the CRS approach; this result is consistent with previous findings [25,26,44]; however, the AE score varied.

The average TE scores based on the assumption of CRS and VRS were 0.82 and 0.90, respectively. Cocoa farms with TE scores exceeding 0.90 based on the assumption of CRS and VRS were 36.56% and 64.15%, respectively. The majority of cocoa farms yielded TE scores between 0.70 and 1.00, under the CRS and VRS assumptions, while fewer than 18% of cocoa farms had a TE score less than 0.70. The average AE scores based on the assumption of CRS and VRS were 0.46 and 0.45, respectively. Cocoa farms with AE scores greater than 0.90 based on the assumption of CRS and VRS were 2.12% and 3.30%, respectively. The majority of cocoa farms had an AE score less than 0.70 based on the two assumptions. Further, fewer than 16% of cocoa farms had an AE score exceeding 0.70. The average EE scores based on the assumption of CRS and VRS were 0.38 and 0.41, respectively. This implies that cocoa farms in Indonesia are not operating with EE. Cocoa farms with EE scores higher than 0.90 based on the assumptions of CRS and VRS accounted for 1.42% and 3.07%, respectively. The majority of cocoa farms had EE scores less than 0.60 (84% for CRS and 80% for VRS).

The average SE was 0.92; this indicates that 8.4% of cocoa farms’ costs could be eliminated. Reductions in the cost of inputs that do not reduce the output can increase the net profit of cocoa farms. One of the ways to accomplish this is to change the scale of cocoa farms and to implement the recommended technology. The majority of cocoa farms (72.64%) had SE scores greater than 0.90. Fewer than 27% of cocoa farms had an SE between 0.5 and 0.90, and fewer than 2% of cocoa farms had an SE less than 0.50. On average, the EE and AE scores showed it is possible to reduce the cost of variable inputs in cocoa farms. Adjustments in the cocoa farm scale would offer the scope to increase efficiency. A significant cost reduction could be achieved by moving cocoa farm production toward the frontier isoquant through the efficient use (TE) and reallocation of inputs (allocative efficiency). The frontier isoquant represents the production possibilities frontiers; the points on the frontier isoquant describe the efficient production levels because all resources would be exhaustively utilized in this position [45].

### Estimation parameters of the factors that affected TE, AE, and EE

Estimated parameters of the factors that affected TE, AE, and EE based on the Tobit model are presented in Table 6.

**Table 6:** Factors that affected TE, AE, and EE of cocoa farms.

Model	TE		AE		EE	
	Estimate	Std. error	Estimate	Std. error	Estimate	Std. error
Intercept	0.83	0.00	0.46	0.01	0.39	0.00
Seed type	0.02***	0.00	0.05***	0.01	0.05***	0.00
Use of organic fertilizers	0.05***	0.01	0.17***	0.01	0.16***	0.01
Extension and training	0.01*	0.00	0.02***	0.01	0.02***	0.00
Access to credit	0.07***	0.00	0.05***	0.01	0.07***	0.00
Access to market	0.12***	0.00	0.01***	0.01	0.04***	0.00
Participation of women	0.03***	0.00	0.01^ns^	0.01	0.02***	0.00
Gender of cocoa farm manager	0.01*	0.01	0.03***	0.01	0.03***	0.01
Sigma	0.08***	0.00	0.12***	0.00	0.08***	0.00
Log likelihood	373.87		298.26		447.04	

Note: TE = overall TE, AE = allocative efficiency, EE = economic efficiency,

*** Significant at 1%,

* Significant at 15%, ns = non significant

Table 6 demonstrates a positive and significant relationship of independent variables based on TE, AE, and EE. The results indicate inefficiency in the production of the majority of cocoa farms, in relation to the variables presented in Table 6. The type of seeds used by farmers had a significant and positive impact on TE, AE, and EE. This shows that superior varieties increase technical, allocative, and economic efficiencies of cocoa farms, resulting in higher productivity. Superior varieties could play an important role in increasing income and overcoming poverty in rural communities. The use of organic fertilizers also had a significant and positive impact on TE, AE, and EE. This implies that the organic material increased technical, allocative, and economic efficiencies of cocoa farms. The use of organic fertilizers can potentially increase the TE, resulting in higher productivity [46]. Organic fertilizers could also play an important role in preserving the ecosystem and increasing the availability of carbon (C) and nitrogen (N) [47]. Organic fertilizers could increase soil fertility; the use of organic fertilizers in farming could increase the soil C content by 2.2% every year [48]. This shows that organic fertilizers could increase the efficiency of chemical fertilizer absorption by cocoa plants, increasing the productivity and EE of cocoa farming. A lower cocoa farming efficiency (TE) was observed for farmers who used chemical fertilizers and did not use organic fertilizers.

The number of extension and training practices followed by farm managers also had a significant and positive impact on the TE, AE, and EE. Again, this implies that extension and training could increase the technical, allocative and economic efficiencies of cocoa farms because extension and training activities play a major role in disseminating technology among farmers. Extension and training have the potential to increase the TE, resulting in higher productivity. Extension and training help farmers with decision making, especially with respect to the use of superior varieties, agricultural cultivation, and access to the cocoa market. Extension and training are related to farm management skills, indicating that these factors could increase overall farm efficiency [49–51]. These factors could contribute to the income of farmers and thus reduce poverty.

Access to credit had a significant and positive impact on TE, AE, and EE. This indicates that access to credit could increase the technical, allocative, and economic efficiencies of cocoa farms. Access to credit can enable farmers to obtain the required farming inputs.

Adequate market access had further significant and positive impacts on the TE and EE, but its impact was not significant for AE. This finding indicates that access to market could increase the technical and EE scores of cocoa farms. Small-scale cocoa farmers in rural areas are often limited by market access due to a lack of information about the cocoa market and ineffective marketing chains. This results in reduced income, and thus the availability of production inputs is also compromised. An adequate and effective marketing market structure (direct marketing or a cooperative approach) will help farmers sell their products, and ultimately, would increase their income and reduce poverty in rural areas.

Women’s participation had a significant and positive impact on the TE, AE, and EE; it was further observed that women’s participation could increase the technical, allocative, and economic efficiencies of cocoa farms. Furthermore, the farm managers’ gender also had a significant impact on TE, AE, and EE. It was also found that women managers were more efficient than men managers. Women’s participation in cocoa farms included the following areas: sanitation or plant management, harvesting, post-harvest activities, and marketing. Women farmers were an indispensable part of the farm management and boosted the quality of cocoa beans (fermentation), which shows that their contribution could increase the income and help in reducing poverty in rural areas. This would ensure the sustainability of cocoa farming inputs, which would consequently affect the TE, AE, and EE.

### Results of the analysis cost reduction potential of cocoa farms

The results of the analysis demonstrate that cocoa farming in the research area is inefficient. Information from the composition of costs is extremely important for the development of effective policies and consequently, an increase in efficiency. The average efficiency of the economy, actual cost, minimum cost, and cost reduction potential of cocoa farms are presented in Table 7.

**Table 7:** Cost reduction potential of cocoa farms.

Variables	n	Mean economic efficiency	Actual cost (IDR)	Minimum cost (IDR)	Reduction cost (IDR)	Reduction cost (%)
Cost minimization by seed type						
Local seed	236	0.24	18,526,039	4,246,689	14,279,350	75.97
Quality seed	188	0.56	17,055,035	9,510,327	7,544,708	43.55
t-value (local seed vs. quality seed)		–23.76***	1.97*	–14.58***	11.80***	
Cost minimization by organic fertilizer						
Non-organic fertilizer	252	0.31	17,199,566	4,857,760	12,341,806	69.38
Organic fertilizer	172	0.50	18,861,636	9,104,679	9,756,957	50.20
t-value (non-organic fertilizer vs. organic fertilizer)		–9.92***	–2.29**	–10,416***	3.95***	
Cost minimization by extension and training						
Following extension and training 0–5 times	310	0.33	17,196,063	5,491,129	11,704,935	68.07
Following extension and training 6–10 times	114	0.64	17,275,188	10,913,279	6,361,910	36.17
t-value (0–5 times vs. 6–10 times)		–2.47***	1.11	–13.37***	12.87***	
Cost minimization by access to credit						
Non-bank credit	200	0.29	17,845,662	4,824,580	13,021,082	71.03
Bank credit	224	0.47	17,928,684	8,156,025	9,772,659	53.16
t-value (non-bank credit vs. bank credit)		–9.88***	–0.16	–8.74***	4.81***	
Cost minimization by access to market						
Access to non-market	201	0.29	17,764,055	4,804,114	12,959,941	71.04
Access to market	223	0.47	17,972,721	8,181,763	9,790,958	53.09
t-value (access to non-market vs. access to market)		–9.98***	–0.32	–8.89***	4.73***	
Cost minimization by participation of women						
Non-participation of women	226	0.28	18,157,271	4,842,368	13,314,903	71.85
Participation of women	198	0.50	17,550,246	8,564,571	8,985,675	49.89
t-value (non-participation of women vs. participation of women)		–2.46***	0.78	–9.78***	6.77***	
Cost minimization by gender of manager						
Men managers	226	0.28	18,116,518	4,824,158	13,292,360	71.82
Women managers	198	0.50	17,596,762	8,585,356	9,011,406	49.93
t-value (men vs. women)		–12.40***	0.66	–9.65***	6.69***	
The average cost reduction						59.65

Note: n = number of sample farms,

*** significant at 1%,

** significant at 5%,

* significant at 10%

The minimum costs indicate the amount of costs that could be spent on farms if the farms operate at the frontier with a certain price level and fixed supporting factors. The minimum cost is obtained by multiplying the actual cost with the EE scores of each farm. The cost reduction potential refers to the value lost due to technical and allocative inefficiency of cocoa farms. The cost reduction potential is calculated by multiplying the actual cost by the index of inefficiency [43].

Table 7 reveals that cocoa farms would be able to reduce their actual costs by 60% if the farms’ production is operated on the frontier. The use of quality seeds and extension and training yielded higher EE levels than other variables. Farm managers who often included extension and training experienced the best cost reduction potential due to their higher EE. Cocoa farms that used organic fertilizers had higher EE compared to those who did not use organic fertilizers, and managers who had access to credit from banks as well as access to the market performed better than those who had no credit. This implies that the use of organic fertilizers, credit programs, and access to market can have a positive impact on cocoa farms. Cocoa farms in which women participated or those that were managed by women showed higher EE levels and the best potential for cost reduction. This suggests that cocoa farm efficiency could be increased by empowering women farmers, providing them non-formal education, such as extension and training, along with capacity building programs, as well as increasing the amount of resources provided to them and improving their access to assets [52,53]. Involving women in farming can increase the production, income, and inventory of household assets. In addition, women can also improve control over farm production, income, and assets [54].

## Conclusion

The results reveal that the majority of cocoa farmers operate their farms relatively inefficiently. The average technical and allocative efficiencies were 0.818 and 0.462, respectively. This implies that there is potential for improving the technical and allocative efficiencies of cocoa farms; the cost reduction potential ranged from 36 to 76%, with an average of 60%. The cost reduction could be achieved through the adoption of the best technologies and resources owned by the farmers. An analysis of the Tobit model demonstrated that the technical and allocative efficiencies of cocoa farm economies are affected by the use of quality seeds, organic fertilizers, frequency of extension and training of farm managers, access to credit from banks, access to markets, participation of women, and farm managers’ gender. This implies that all these variables are important for increasing the output. The increased output would increase farmers’ income and reduce poverty in rural areas. Policymakers need to consider these variables. Given that the role of women in cocoa production is essential, this research recommends the formulation of a policy that promotes women’s capabilities (such as extension and training and support for women farmers’ groups). Extension and training are needed to increase the frequency, content, and quality, as these factors could contribute to an increase in efficiency. Extension and training could also reduce the costs associated with technical and allocative inefficiencies such that the productivity and income of cocoa farms could increase. Policymakers need to develop programs that render production credit more easily accessible to farmers, especially through cooperatives and banks.

## Supporting information

S1 File Kuesioner 2016. (DOC)

S2 File Questionnaire 2016. (DOCX)

S3 File DATA. (XLS)
