# Supplementary material for: Factors influencing the efficiency of cocoa farms: A study to increase income in rural Indonesia
Source: PLoS One. 2019 Apr 4;14(4):e0214569. doi: 10.1371/journal.pone.0214569 (PMC6448898; doi:10.1371/journal.pone.0214569)
Supplement: S1 File — (DOC) [file pone.0214569.s001.doc]

PERAN DAN TUGAS TENAGA LAPANGAN

| **NO.** | **JENIS PERAN** | **TUGAS** |
| --- | --- | --- |
| 1 | Enumerator | Berkoordinasi dengan fasilitator lapangan dalam penentuan  narasumber (responden) di desa untuk diwawancara |
|  |  | Melakukan wawancara terhadap responden |
|  |  | Berkoordinasi dengan supervisor dalam pengisian kuesioner |
| 2 | Supervisor | Mereview dan mengasistensi pengisian kuesioner yang  dilakukan oleh enumerator dan memastikan kuesioner terisi  dengan benar (valid) |
|  |  | Mengimput data kuesioner yang telah diisi oleh responden  dan telah divalidasi datanya |
|  |  | Memonitoring seluruh kegiatan Enumerator yang berada  dibawah clusternya |
|  |  | Berkoordinasi dengan tim tenaga ahli dalam pelaksanaan  survei dan jika terjadi permasalahan di lapangan |
| 3 | Fasilitator lapangan/Desa | Membantu dalam memobilisasi Enumerator dan Supervisor  di lapangan |
|  |  | Membantu dalam perizinan pelaksanaan survei di lapangan |
|  |  | Berkoordinasi dengan enumerator dalam penentuan  responden untuk diwawancara |

**PANDUAN PELAKSANAAN SURVEI**

| **NO.** | **URAIAN** | **KETERANGAN** |
| --- | --- | --- |
| 1 | Lokasi | Cluster Donggala 1: Desa Watatu dan Salumpaku |
|  |  | Cluster Parigi Moutong 2: Desa Kota Raya dan Kayu Agung |
|  |  | Cluster Sigi 3: Desa Sejahtera dan Tongoa |
|  |  | Cluster Poso 4: Desa Lape dan Kilo |
|  |  | Setiap Enumerator menempati satu desa |
|  |  | Setiap satu cluster didampingi oleh satu supervisor |
| 2 | Responden | Pengambilan sampel dilakukan secara acak |
|  |  | Jumlah responden ditentukan sebagai berikut: |

Penentuan ukuran sampel dihitung dengan menggunakan rumus Parel et al., (1973), dengan rumus penentuan jumlah sampel sebagai berikut:

$n=\frac{N\sum N_{h}s_{h}^{2}}{N^{2}\frac{d^{2}}{z^{2}}+\sum N_{h}s_{h}^{2}}$ (1)

dimana : n = Jumlah Sampel

N = Jumlah populasi

N_h_ = Jumlah populasi dalam tiap desa

d = Presisi yang ditetapkan sebesar = 10%

z = 1,645 (90%)

s_h_ = varian dari masing-masing desa

Penentuan sampel dari tiap-tiap desa ditentukan secara proposional dalam 1 cluster dengan rumus sebagai berikut.

$n_{h}=\frac{N_{h}}{N}n$ (2)

dimana:

n_h_ = Jumlah sampel dalam desa

Hasil perhitungan jumlah sampel masing-masing desa terlihat pada Tabel 1, 2, 3, dan 4.

**Tabel 1. Cluster Donggala**

|  |  |  |  |  |  |  |  |
| --- | --- | --- | --- | --- | --- | --- | --- |
| DESA | Nh | Sh | Sh2 | Nh*Sh2 | n | nh | nh bulat |
| Watatu | 65 | 0.52 | 0.27 | 17.70 |  | 46.17 | 46 |
| Salumpaku | 58 | 0.53 | 0.29 | 16.56 |  | 41.19 | 41 |
| N | 123 |  |  |  |  | 87.36 |  |
| **Jumlah** |  |  |  | 34.26 | 87.36 |  | **87** |

**Tabel 2. Cluster Parigi Moutong**

|  |  |  |  |  |  |  |  |
| --- | --- | --- | --- | --- | --- | --- | --- |
| DESA | Nh | Sh | Sh2 | Nh*Sh2 | n | nh | nh bulat |
| Kota Raya | 72 | 0.58 | 0.34 | 24.48 |  | 53.74 | 54 |
| Kayu Agung | 59 | 0.61 | 0.38 | 22.19 |  | 44.04 | 44 |
| N | 131 |  |  |  |  | 97.78 |  |
| **Jumlah** |  |  |  | 46.67 | 97.78 |  | **98** |

**Tabel 3. Cluster Sigi**

|  |  |  |  |  |  |  |  |
| --- | --- | --- | --- | --- | --- | --- | --- |
| DESA | Nh | Sh | Sh2 | Nh*Sh2 | n | nh | nh bulat |
| Sejahtera | 106 | 0.55 | 0.31 | 32.64 |  | 64.07 | 64 |
| Tongoa | 132 | 0.60 | 0.36 | 47.31 |  | 79.78 | 80 |
| N | 238 |  |  |  |  | 143.85 |  |
| **Jumlah** |  |  |  | 79.95 | 143.85 |  | **144** |

**Tabel 4. Cluster Poso**

|  |  |  |  |  |  |  |  |
| --- | --- | --- | --- | --- | --- | --- | --- |
| DESA | Nh | Sh | Sh2 | Nh*Sh2 | n | nh | nh bulat |
| Lape | 77 | 0.54 | 0.29 | 22.68 |  | 52.11 | 52 |
| Kilo | 63 | 0.49 | 0.24 | 15.23 |  | 42.63 | 43 |
| N | 140 |  |  |  |  | 94.74 |  |
| **Jumlah** |  |  |  | 37.90 | 94.74 |  | **95** |

| 3 | Mekanisme Pengumpulan Data | Enumerator berkoordinasi dengan Fasilitator Lapangan  dalam penentuan responden (penganbilan sampel  secara acak) |
| --- | --- | --- |
|  |  | Enumerator berkoordinasi dengan Supervisor dalam  pengisian kuesioner untuk memperoleh data yang valid.  Hasil pengisian kuesioner diasistensi oleh Supervisor  sebelum diliput. |

**PENGISIAN KUESIONER**

**CLUSTER ……….**

**NAMA DESA ……….**

**NAMA ENUMERATOR ……….**

**Bapak dan Ibu responden yang terhormat**

Dengan segala kerendahan hati kami mohon kesediaan Bapak/Ibu untuk mengisi dan memberikan jawaban sesuai dengan keadaan dan kenyataan yang sebenarnya. Terima kasih atas kesediaan Bapak/ibu menjadi responden dalam penelitian dan telah mengisi kuisioner ini, semoga Tuhan Yang Maha Esa akan membalasNya, Amin.

1. Nomor Responden : ………………

2. Jenis Kelamin : a. Laki-Laki

b. Perempuan

3. Umur : ………………Tahun

4. Pekerjaan Utama : a. Petani

b. PNS

c . ABRI

d. Polisi

e. Karyawan

f. Wiraswasta

5. Pendidikan : a. Tidak Tamat SD

b. Tamat SD

c. Tamat SMP

d. Tamat SMA

e. Tamat PT

6. Pengalaman berusahatani kakao : ....................... Tahun

7. Jenis bibit yang digunakan : a. Dari kebun petani lokal (tidak berlabel)

b. bibit terpilih yang dikeluarkan oleh departemen

pertanian (berlabel)

8. Berapa kali bapak/ibu mengikuti penyuluhan dan pelatihan usahatani kakao? ...............

9. Apakah bapak/ibu mengerti dengan materi yang diberikan dalam penyuluhan dan pelatihan usahatani kakao?

a. Ya

b. Tidak

10. Apakah bapak/ibu dalam berusahatani kakao menggunakan fasilitas kredit?

a. Ya

b. Tidak

11. Jika Ya, fasilitas kredit apa yang bapak/ibu gunakan:

a. Bank pemerintah

b. Bank swasta

c. Non-bank

12. Berapa besar nilai kredit yang bapak/ibu peroleh/tahun Rp...............................

13. Dari mana bapak/ibu mengetahui harga biji kakao:

a. Dari pedagang besar melalui HP

b. Tidak mengetahui

14. Dimana bapak/ibu menjual biji kakao:

a. Pedagang pengumpul desa

b. Pedagang besar kabupaten

15. Berapa upah tenaga kerja sebagai buruh tani di desa bapak/ibu

Rp …............................................. / hari orang kerja (HOK)

16. Biaya Usahatani:

A. BIAYA TETAP (TFC)

| URAIAN | SATUAN | HARGA/UNIT | TGL PEMBELIAN | LAMA PEMAKAIAN (TAHUN) |
| --- | --- | --- | --- | --- |
| 1.Luas Lahan Garapan | ………ha | ………. (Pajak/thn) | xxxxxxxxxxxx | xxxxxxxxxxx |
| 2.Cangkul | ………bh | ………. | ………. | ………… |
| 3.Cangkul garpu | ………bh | ………. | ………. | ………… |
| 4.Golok | ………bh | ………. | ………. | ………… |
| 5.Sabit | ………bh | ………. | ………. | ………… |
| 6.Hand sprayer | ………bh | ………. | ………. | ………… |
| 7.Terpal | ………bh | ………. | ………. | ………… |
| 8.Pisau | …… bh | ………. | ………. | ………… |
| 9……………………. | ………bh | ………. | ………. | ………… |
| 10………………. | …… bh | ………. | ………. | ………… |

B. BIAYA VARIABEL (TVC), Sesuai dengan Luas Lahan yang diusahakan

| URAIAN | SATUAN (kg) | HARGA/UNIT |
| --- | --- | --- |
| 1.PUPUK: |  |  |
| a. Urea | ……… | ………. Rp/kg |
| b. SP 36 | ……… | ………. Rp/kg |
| c. KCl | ……… | ………. Rp/kg |
| D Organik | ……… | ………. Rp/kg |
| e……………………. | ……… | ………. Rp/kg |
|  |  |  |
|  |  |  |
| 2.PESTISIDA: | SATUAN (Lt) | HARGA/UNIT |
| a……………………. | ……… | ……….Rp/Lt |
| b…………………….. | ……… | ………. Rp/Lt |
| c…………………….. | ……… | ………. Rp/Lt |
| d…………………….. | ……… | ………. Rp/Lt |
| e……………………… | ……… | ………. Rp/Lt |
|  |  |  |
|  |  |  |

C. TENAGA KERJA (TK)

1. Penyiangan lahan

| No. | Jenis TK | Satuan (jam/hari/orang) | Jumlah tenaga kerja (orang) | Jumlah hari kerja  (hari) | Upah /hari/orang (Rp) |
| --- | --- | --- | --- | --- | --- |
| 1. | TK Pria |  |  |  |  |
| 2. | TK Wanita |  |  |  |  |
| Jumlah | |  |  |  |  |

Keterangan : Semua TK diasumsikan dari luar keluarga (upah diperhitungkan).

*2. Pemangkasan pohon kakao*

| No. | Jenis TK | Satuan (jam/hari/orang) | Jumlah tenaga kerja (orang) | Jumlah hari kerja  (hari) | Upah /hari/orang (Rp) |
| --- | --- | --- | --- | --- | --- |
| 1. | TK Pria |  |  |  |  |
| 2. | TK Wanita |  |  |  |  |
| Jumlah | |  |  |  |  |

Keterangan : Semua TK diasumsikan dari luar keluarga (upah diperhitungkan).

*3*. Pemupukan

| No. | Jenis TK | Satuan (jam/hari/orang) | Jumlah tenaga kerja (orang) | Jumlah hari kerja  (hari) | Upah /hari/orang (Rp) |
| --- | --- | --- | --- | --- | --- |
| 1. | TK Pria |  |  |  |  |
| 2. | TK Wanita |  |  |  |  |
| Jumlah | |  |  |  |  |

Keterangan : Semua TK diasumsikan dari luar keluarga (upah diperhitungkan).

*4*. Pemberantasan hama dan penyakit

| No. | Jenis TK | Satuan (jam/hari/orang) | Jumlah tenaga kerja (orang) | Jumlah hari kerja  (hari) | Upah /hari/orang (Rp) |
| --- | --- | --- | --- | --- | --- |
| 1. | TK Pria |  |  |  |  |
| 2. | TK Wanita |  |  |  |  |
| Jumlah | |  |  |  |  |

Keterangan : Semua TK diasumsikan dari luar keluarga (upah diperhitungkan).

*5*. Pemetikan dan pengumpulan buah kakao

| No. | Jenis TK | Satuan (jam/hari/orang) | Jumlah tenaga kerja (orang) | Jumlah hari kerja  (hari) | Upah /hari/orang (Rp) |
| --- | --- | --- | --- | --- | --- |
| 1. | TK Pria |  |  |  |  |
| 2. | TK Wanita |  |  |  |  |
| Jumlah | |  |  |  |  |

Keterangan : Semua TK diasumsikan dari luar keluarga (upah diperhitungkan).

*6. Pengangkutan hasil panen kakao (dari lahan kerumah petani)*

| No. | Jenis TK | Satuan (jam/hari/orang) | Jumlah tenaga kerja (orang) | Jumlah hari kerja  (hari) | Upah /hari/orang (Rp) |
| --- | --- | --- | --- | --- | --- |
| 1. | TK Pria |  |  |  |  |
| 2. | TK Wanita |  |  |  |  |
| Jumlah | |  |  |  |  |

Keterangan : Semua TK diasumsikan dari luar keluarga (upah diperhitungkan).

*7.* Pengupasan buah kakao

| No. | Jenis TK | Satuan (jam/hari/orang) | Jumlah tenaga kerja (orang) | Jumlah hari kerja  (hari) | Upah /hari/orang (Rp) |
| --- | --- | --- | --- | --- | --- |
| 1. | TK Pria |  |  |  |  |
| 2. | TK Wanita |  |  |  |  |
| Jumlah | |  |  |  |  |

Keterangan : Semua TK diasumsikan dari luar keluarga (upah diperhitungkan).

*8.* Penjemuran biji kakao

| No. | Jenis TK | Satuan (jam/hari/orang) | Jumlah tenaga kerja (orang) | Jumlah hari kerja  (hari) | Upah /hari/orang (Rp) |
| --- | --- | --- | --- | --- | --- |
| 1. | TK Pria |  |  |  |  |
| 2. | TK Wanita |  |  |  |  |
| Jumlah | |  |  |  |  |

Keterangan : Semua TK diasumsikan dari luar keluarga (upah diperhitungkan).

17. Produksi:

| Jenis Tanaman | Luas Areal Panen (ha) | Jumlah Pohon Kakao | Jarak Tanam  (m x m) | Produksi (kg) | Potongan produksi (%) atau (kg) | Harga (Rp/kg) |
| --- | --- | --- | --- | --- | --- | --- |
| 1. Kakao |  |  |  |  |  |  |
