# Supplementary material for: Factors influencing the efficiency of cocoa farms: A study to increase income in rural Indonesia
Source: PLoS One. 2019 Apr 4;14(4):e0214569. doi: 10.1371/journal.pone.0214569 (PMC6448898; doi:10.1371/journal.pone.0214569)
Supplement: S2 File — (DOCX) [file pone.0214569.s002.docx]

**THE ROLES AND TASKS OF FIELD LABOR**

| **NUMBER** | **TYPES OF ROLES** | **TASKS** |
| --- | --- | --- |
| 1 | Enumerator | Coordinate with field facilitators in the determination  of resource persons (respondents) in the village to  be interviewed |
|  |  | Conducting interviews with respondents |
|  |  | Coordinate with the supervisor in filling out the  questionnaires |
| 2 | Supervisor | Review and help filling in the questionnaires done  by the enumerator and ensure the questionnaires are  filled correctly (valid) |
|  |  | Input data questionnaires that have been filled by the  respondents and have validated its data |
|  |  | Monitoring all activities of Enumerator under which  are under its cluster |
|  |  | Coordinate with a team of experts in conducting the  survey and in case of problems in the field |
| 3 | Field / Village Facilitator | Assist in mobilizing Enumerators and Supervisors  in the field |
|  |  | Assist in licensing of survey implementation in  the field |
|  |  | Coordinate with the enumerator in determination  the respondents for the interview |

**SURVEY IMPLEMENTATION GUIDE**

| **NUMBER** | **DESCRIPTION** | **INFORMATION** |
| --- | --- | --- |
| 1 | Location | Cluster Donggala 1: Watatu and Salumpaku Village |
|  |  | Cluster Parigi Moutong 2: Kota Raya and  Kayu Agung Village |
|  |  | Cluster Sigi 3: Sejahtera and Tongoa Village |
|  |  | Cluster Poso 4: Lape and Kilo Village |
|  |  | Each Enumerator occupies one village |
|  |  | Each cluster is accompanied by one supervisor |
| 2 | Respondents | Sampling is done randomly |
|  |  | The number of respondents are determined as follows: |

Determination of sample size is calculated using Parel et al., (1973), with the formula for determining number of sample as follows:

$n=\frac{N\sum N_{h}s_{h}^{2}}{N^{2}\frac{d^{2}}{z^{2}}+\sum N_{h}s_{h}^{2}}$ (1)

where : n = Number of sample

N = Jumlah populasi

N_h_ = Number of population in each village

d = Precision set at = 10%

z = 1,645 (90%)

s_h_ = variant of each village

The sample determination of each village is determined proportionally in 1 cluster with the following formula.

$n_{h}=\frac{N_{h}}{N}n$ (2)

where:

n_h_ = Number of sample in the village

The calculation results of the samples numbers of each village are shown in Table 1, 2, 3, and 4.

| **Table 1. Cluster Donggala** |  |  |  |  |  |  |  |
| --- | --- | --- | --- | --- | --- | --- | --- |
| VILLAGE | Nh | Sh | Sh2 | Nh*Sh2 | n | nh | nh round |
| Watatu | 65 | 0.52 | 0.27 | 17.70 |  | 46.17 | 46 |
| Salumpaku | 58 | 0.53 | 0.29 | 16.56 |  | 41.19 | 41 |
| N | 123 |  |  |  |  | 87.36 |  |
| **Total** |  |  |  | 34.26 | 87.36 |  | **87** |

**Table 2. Cluster Parigi Moutong**

|  |  |  |  |  |  |  |  |
| --- | --- | --- | --- | --- | --- | --- | --- |
| VILLAGE | Nh | Sh | Sh2 | Nh*Sh2 | n | nh | nh round |
| Kota Raya | 72 | 0.58 | 0.34 | 24.48 |  | 53.74 | 54 |
| Kayu Agung | 59 | 0.61 | 0.38 | 22.19 |  | 44.04 | 44 |
| N | 131 |  |  |  |  | 97.78 |  |
| **Total** |  |  |  | 46.67 | 97.78 |  | **98** |

**Table 3. Cluster Sigi**

|  |  |  |  |  |  |  |  |
| --- | --- | --- | --- | --- | --- | --- | --- |
| VILLAGE | Nh | Sh | Sh2 | Nh*Sh2 | n | nh | nh round |
| Sejahtera | 106 | 0.55 | 0.31 | 32.64 |  | 64.07 | 64 |
| Tongoa | 132 | 0.60 | 0.36 | 47.31 |  | 79.78 | 80 |
| N | 238 |  |  |  |  | 143.85 |  |
| **Total** |  |  |  | 79.95 | 143.85 |  | **144** |

**Tabel 4. Cluster Poso**

|  |  |  |  |  |  |  |  |
| --- | --- | --- | --- | --- | --- | --- | --- |
| VILLAGE | Nh | Sh | Sh2 | Nh*Sh2 | n | nh | nh round |
| Lape | 77 | 0.54 | 0.29 | 22.68 |  | 52.11 | 52 |
| Kilo | 63 | 0.49 | 0.24 | 15.23 |  | 42.63 | 43 |
| N | 140 |  |  |  |  | 94.74 |  |
| **Total** |  |  |  | 37.90 | 94.74 |  | **95** |

| 3 | Data Collection Mechanism | The Enumerator coordinates with the Field Facilitator in  the determination of respondents (sampling done randomly) |
| --- | --- | --- |
|  |  | The Enumerator coordinates with the Supervisor in filling  the questionnaires to obtain the valid data.  The filling results of questionnaires are supervised by the  Supervisor before they are covered. |

**QUESTIONNAIRE FILLING**

**CLUSTER ……….**

**NAME OF VILLAGE ……….**

**NAME OF ENUMERATOR ……….**

**Dear Mr. and Mrs. Respondent**

With all humility, we ask your willingness to fill and give answers according to the actual circumstances and reality. Thank you for your willingness to be a respondent in the research and have filled out this questionnaire, may God Almighty will repay it, Amen.

1. Number of Respondent : ………………

2. Gender : a. Male

b. Female

3. Age : ………………Years Old

4. Main job : a. Farmers

b. Civil servants

c . Indonesian National Armed Forces

d. Police

e. Employe

f. Entrepreneur

5. Education : a. Not Graduated from primary school

b. Graduated from primary school

c. Graduated from junior high school

d. Graduated from high school

e. Graduated from high college

6. Experience of cocoa farming : ....................... Years

7. Type of seed used : a. From local farmers' farm (not labeled)

b. selected seeds released by the Ministry of

Agriculture (labeled)

8. How many times do you follow the extension and training of cocoa farming? ...............

9. Do you understand the material given in extension and training of cocoa farming?

a. Yes

b. No

10. Do you use credit facilities in cocoa farming?

a. Yes

b. No

11. If yes, what credit facility do you use :

a. Government Bank

b. Private Bank

c. Non-bank

12. How much credit value you get / year? IDR...............................

13. Where do you know the price of cocoa beans:

a. From wholesalers through Mobile Phone

b. Do not know

14. Where do you sell cocoa beans:

a. Village collectors traders

b. Great district trader

15. How much is the wage of labor as a farm laborer in your village

IDR …............................................. / day people work (DPW)

16. Farming Costs:

A. FIXED COSTS (TFC)

| DESCRIPTION | UNIT | PRICE/UNIT | PURCHASE DATE | LONG USAGE (YEAR) |
| --- | --- | --- | --- | --- |
| 1. Cultivated Land Area | ………ha | ………. (Tax / year) | xxxxxxxxxxxx | xxxxxxxxxxx |
| 2. Hoe | ……… piece | ………. | ………. | ………… |
| 3. Hoe fork | ……… piece | ………. | ………. | ………… |
| 4. Machete | ……… piece | ………. | ………. | ………… |
| 5. Sickle | ……… piece | ………. | ………. | ………… |
| 6. Hand sprayer | ……… piece | ………. | ………. | ………… |
| 7. Tarpaulins | ……… piece | ………. | ………. | ………… |
| 8. Knife | …… piece | ………. | ………. | ………… |
| 9……………………. | ……… piece | ………. | ………. | ………… |
| 10………………. | …… piece | ………. | ………. | ………… |

B. VARIABLE COSTS (TVC), In accordance with the area of land cultivated

| DESCRIPTION | UNIT (kg) | PRICE/UNIT |
| --- | --- | --- |
| 1. FERTILIZER: |  |  |
| a. Urea | ……… | ………. IDR/kg |
| b. SP 36 | ……… | ………. IDR/kg |
| c. KCl | ……… | ………. IDR/kg |
| d. Organic | ……… | ………. IDR/kg |
| e……………………. | ……… | ………. IDR/kg |
|  |  |  |
|  |  |  |
| 2. PESTICIDE: | UNIT (Lt) | PRICE/UNIT |
| a……………………. | ……… | ……….IDR/Lt |
| b…………………….. | ……… | ………. IDR/Lt |
| c…………………….. | ……… | ………. IDR/Lt |
| d…………………….. | ……… | ………. IDR/Lt |
| e……………………… | ……… | ………. IDR/Lt |
|  |  |  |
|  |  |  |

C. LABOR

*1. Weeding/sanitation Land*

| Number | Type of Labor | Unit (hour / day / people) | Number of labor (people) | Number of working days (days) | Wage / day / people (IDR) |
| --- | --- | --- | --- | --- | --- |
| 1. | Men labor |  |  |  |  |
| 2. | Women labor |  |  |  |  |
| Total | |  |  |  |  |

Description: All laborers are assumed from outside the family (wages are taken into account).

*2. Pruning cocoa trees*

| Number | Type of Labor | Unit (hour / day / people) | Number of labor (people) | Number of working days (days) | Wage / day / people (IDR) |
| --- | --- | --- | --- | --- | --- |
| 1. | Men labor |  |  |  |  |
| 2. | Women labor |  |  |  |  |
| Total | |  |  |  |  |

Description: All laborers are assumed from outside the family (wages are taken into account).

*3. Fertilization*

| Number | Type of Labor | Unit (hour / day / people) | Number of labor (people) | Number of working days (days) | Wage / day / people (IDR) |
| --- | --- | --- | --- | --- | --- |
| 1. | Men labor |  |  |  |  |
| 2. | Women labor |  |  |  |  |
| Total | |  |  |  |  |

Description: All laborers are assumed from outside the family (wages are taken into account).

*4. Eradication of pests and diseases*

| Number | Type of Labor | Unit (hour / day / people) | Number of labor (people) | Number of working days (days) | Wage / day / people (IDR) |
| --- | --- | --- | --- | --- | --- |
| 1. | Men labor |  |  |  |  |
| 2. | Women labor |  |  |  |  |
| Total | |  |  |  |  |

Description: All laborers are assumed from outside the family (wages are taken into account).

*5. Picking and collecting cocoa fruits*

| Number | Type of Labor | Unit (hour / day / people) | Number of labor (people) | Number of working days (days) | Wage / day / people (IDR) |
| --- | --- | --- | --- | --- | --- |
| 1. | Men labor |  |  |  |  |
| 2. | Women labor |  |  |  |  |
| Total | |  |  |  |  |

Description: All laborers are assumed from outside the family (wages are taken into account).

*6. Transportation of cocoa harvest (from land to farmer's house)*

| Number | Type of Labor | Unit (hour / day / people) | Number of labor (people) | Number of working days (days) | Wage / day / people (IDR) |
| --- | --- | --- | --- | --- | --- |
| 1. | Men labor |  |  |  |  |
| 2. | Women labor |  |  |  |  |
| Total | |  |  |  |  |

Description: All laborers are assumed from outside the family (wages are taken into account).

*7. Peeling the cocoa fruit*

| Number | Type of Labor | Unit (hour / day / people) | Number of labor (people) | Number of working days (days) | Wage / day / people (IDR) |
| --- | --- | --- | --- | --- | --- |
| 1. | Men labor |  |  |  |  |
| 2. | Women labor |  |  |  |  |
| Total | |  |  |  |  |

Description: All laborers are assumed from outside the family (wages are taken into account).

*8. Drying cocoa beans*

| Number | Type of Labor | Unit (hour / day / people) | Number of labor (people) | Number of working days (days) | Wage / day / people (IDR) |
| --- | --- | --- | --- | --- | --- |
| 1. | Men labor |  |  |  |  |
| 2. | Women labor |  |  |  |  |
| Total | |  |  |  |  |

Description: All laborers are assumed from outside the family (wages are taken into account).

17. Production:

| Type of Plant | Harvest Area (ha) | Number of Cocoa Trees | Distance of Planting (m x m) | Yield  (kg) | Cutting yield (%) or (kg) | Price (IDR/kg) |
| --- | --- | --- | --- | --- | --- | --- |
| 1. Cocoa |  |  |  |  |  |  |
